# Supplementary material for: Genetic insights into the connection between pulmonary TB and non-communicable diseases: An integrated analysis of shared genes and potential treatment targets
Source: PLoS One. 2024 Oct 21;19(10):e0312072. doi: 10.1371/journal.pone.0312072 (PMC11493268; doi:10.1371/journal.pone.0312072)
Supplement: S1 File — This document includes the data sources and accession numbers for each dataset. (DOCX) [file pone.0312072.s001.docx]

**S1 File. Data Availability Statement**

The data used in this study are available from the Gene (<https://www.ncbi.nlm.nih.gov/gene/> ) and GEO datasets (<https://www.ncbi.nlm.nih.gov/geo/> ). The accession numbers for the datasets are for Pulmonary Tuberculosis (PTB) - GSE54992 and GSE19442, Parkinson’s Disease (PD) - GSE20295 and GSE22491, Rheumatoid Arthritis (RA) - GSE23561 and GSE157047. Chronic Kidney Disease (CKD) - GSE66494, GSE15072, and GSE141295. Cerebrovascular Vascular Disease (CVD) - GSE51878 and GSE141910. For Lung Cancer (LC) - GSE42826 and GSE30219. Diabetes Mellitus (DM) - GSE92724 and GSE236746. Additionally, all relevant data are within the manuscript.
