# Supplementary material for: Genetic insights into the connection between pulmonary TB and non-communicable diseases: An integrated analysis of shared genes and potential treatment targets
Source: PLoS One. 2024 Oct 21;19(10):e0312072. doi: 10.1371/journal.pone.0312072 (PMC11493268; doi:10.1371/journal.pone.0312072)
Supplement: Attachment — Submitted filename: Plos one Manuscript_3_5_2024.docx [file pone.0312072.s002.docx]

**I appreciate the opportunity to review this manuscript report that tried to find out the genetic link between Pulmonary TB and Non-communicable diseases.**

**Aims of this study:**

This manuscript investigates the genetic link between Pulmonary Tuberculosis and non-communicable diseases by using in-silico approaches. They tried to find the hub genes and their possible targets that can be utilized as potential drugs for the treatment of these co-morbidities. The authors found nine shared genes between TB and 7 NCDs and five hub genes

They utilized various in-silico approaches to build a genetic link between PTB and 7 NCDs. They have performed various analyses including Protein-protein interactions, Gene regulatory networks, and physical interactions to understand the link between PTB and NCDs and the drug components that can be utilized to treat these co-morbidities. Considering these strengths, I found some areas which require greater clarity and modifications.

**# Specific comments and Recommendations**

**Major Comments:**

1. What is the reason behind the use of the degree method for identifying the hub genes among PTB and NCDs, over other topological analysis methods such as MCC?
2. What are the threshold values of the PPI network used in this paper?
3. Few of the potential hub genes identified in this study require some validation using mRNA expression datasets obtained from PTB and NCD patient tissue samples.
4. To enhance the visual understanding for the readers, it will be a good idea to represent the overlapping genes between PTB and NCDs in a Venn diagram instead of the tabular format shown in Table 3.
5. This manuscript contains numerous typos and grammatical errors which need to be addressed properly by the Authors.
6. The authors touch upon this in their Introduction section, but further justification is required for choosing these 7 NCDs specifically.
7. Briefly describe the limitations of this study and what steps can be taken to address those limitations.

**Minor comments**

8. Rewrite the Objectives section as:

This study aims to identify the drug components that can regulate abnormal gene expression in NCDs. The study will reveal hub genes, potential biomarkers, and drug components associated with hub genes through statistical measures. This will contribute to targeted therapeutic interventions.

9. Rewrite the Results and Conclusions section as:

In this study, we found that all of the hub genes are linked with the 10 drug components, and it was observed that aspirin CTD 00005447 was mostly associated with all the other hub genes. This bio-informatics study may help researchers better understand the cause of PTB and its relationship with NCDs and eventually this can lead to exploring effective treatment plans.

10. Page 4, line 119

Lifestyle, environmental factors, behaviors, and dietary information can cause non-communicable diseases.

Change ‘dietary information” to dietary intakes.

11. Please rewrite/modify the section containing the research gap (Introduction section Para The introduction should be concise and to the point.
12. Page 4, line 122 Rewrite this sentence as” It might be challenging to determine whether or not the diseases are connected because it is difficult to make a straight cause-and-effect inference.”
13. Page 4, line 129 Rewrite this sentence as “some studies included a smaller and narrow dataset wherein the essential risk factors associated with PTB such as smoking, diabetes, socioeconomic status was not taken into consideration.”
14. Page 4 line 132, Rewrite this sentence “This study works with seven non-communicable diseases because, to the best of our knowledge, other studies only worked with four or five non-communicable diseases.”

In this study, we identified /uncovered most of the NCDs as the existing studies have failed to adequately report all of them.

15. Page 4 line 237 “The co-expression and Physical Interaction Network have been accomplished by using GeneMANIA plugin”

Missing keyword “prediction”

The co-expression and Physical Interaction Network prediction was accomplished by using GeneMANIA plugin

16. Page 7, line 244 Rewrite this sentence “It includes gene sets 244 linked to drugs and small molecules based on data about how gene expression changes because of drugs” as: It includes gene sets that relate the drugs and small molecules with the gene expression data obtained upon drug administration.
17. Use the standard scientific nomenclature for gene/protein names throughout the manuscript.

# 18. This paper “An Integrative Network Approach to Identify Common Genes for the Therapeutics in Tuberculosis and Its Overlapping Non-Communicable Diseases” (Alam et al., 2021) should be cited.

19. Page 12, line 299 “Notably, pathways such as Immune System (R-HSA-168256), Cytokine Signaling In Immune System (R-HSA-1280215), and Interleukin-10 Signaling (R-HSA-678378) are also connected with highest number of genes from Reactome pathway”

As per the Reactome pathway analysis, the pathways connected with the highest number of genes as:

1. Signalling By Interleukins R-HAS-449147

2. Cytokine Signaling In Immune System (R-HSA-1280215),

3. Immune system R-HAS-168256
