# Supplementary material for: Genetic insights into the connection between pulmonary TB and non-communicable diseases: An integrated analysis of shared genes and potential treatment targets
Source: PLoS One. 2024 Oct 21;19(10):e0312072. doi: 10.1371/journal.pone.0312072 (PMC11493268; doi:10.1371/journal.pone.0312072)
Supplement: Attachment — Submitted filename: Response_to_Reviewers_PLOS_One.docx [file pone.0312072.s003.docx]

**Response to Reviewers**

**Reviewer #1:**

I wanted to comment upon the language of the paper, I can fully understand it is difficult for a non-native speaker to be fluent in English, but I expect the authors to improve the language of the manuscript thoroughly as the grammatical errors dilute the actual potential of the manuscript.

Author response: Thank you for your insightful comments. We agree with the assessment. We have carefully considered your suggestion and taken actions.

Author action: According to your suggestion, we have improved and corrected the grammar in the manuscript.

**Reviewer 2#:**

Authors have put in a commendable effort using bioinformatics in addressing one of the challenging questions in treatment of patients with comorbidities. In this article authors have investigated the genetic association between PTB and 7 NCDs. They show that genes such as TNF, IL10, NLRP3, IL18, IFNG, HMGB1, CXCL8, IL17A, and NFKB1 discovered were shared between TB and the NCDs, and five hub genes (NFKB1, TNF, CXCL8, NLRP3, and IL10) were found to be common among them. Moreover, they also suggested a few drug candidates for the treatment. Although further investigation is suggested for better treatment outcomes.

The manuscript is very well written.

However, I have one major concern:

Authors have shown NFKB1 as a potential target. Given the pleiotropic role of NFKB1, approach to target NFKB1 rather becomes interesting and the question of specificity immediately arises. It is vital to consider the underlying mechanisms of disease progression to obtain a required beneficial outcome while targeting NFKB1 in patients with comorbidities. Consider following situations involved in comorbidities:

a. In autoimmune diseases like RA immune system needs to be suppressed to alleviate the disease while in PTB need of effective immune system is essential.

b. In cancer, while rouge cells are required to be controlled, immune system needs bolstering.

Also, a more nuanced approach is advised such as targeting of subsets of NF-κB activities which, hopefully, will target pathogenic processes while leaving physiological processes largely intact.

Could the authors discuss the complication of targeting NFKB1, while it is one of the major hub genes as per their study is concerned.

Minor correction

Line no. 378- On the other hand, In tissue-specific PPI, the NFKB1 gene interacts with. Small case, ‘in’.

Author response: Thank you for your insightful comments. We have extensively reviewed the NFKB1 gene. Through this review we identified the complications and subsets of NFKB1. NFKB1 may exacerbate existing complications or cause new ones, worsening symptoms and general well-being in (auto)inflammatory disease. Furthermore, NFKB1 regulates immunological responses. Targeting it may disturb immunological homeostasis, increasing susceptibility to infections or autoimmune disorders.

From a meta-analysis, 94ins/del polymorphism in the NFKB1 promoter is associated with cancer risk, with probable ethnic differences. This emphasizes the relevance of NF-B signaling in oncogenesis and suggests it as a possible therapeutic target.

Author action: According to your suggestion, we have revised and updated the manuscript with blue color in Discussion Section, Page - 18, 19, Reference - [98, 99, 102], Line - (416- 424, 435-438). Additionally, we have made further improvements to the clarity of the language throughout the manuscript, ensuring that each section is described concisely and clearly.

**Specific comments and Recommendations**

**Major Comments:**

1. What is the reason behind the use of the degree method for identifying the hub genes among PTB and NCDs, over other topological analysis methods such as MCC?

Author response: Thank you for your insightful comments. Degree method simply counts the number of direct connections with genes in a network. The degree method is a practical option for initial hub gene identification, especially in large-scale studies, but MCC and other methods are intensive compared to degree method in large network.

Author action: According to your suggestion, we have revised and updated the manuscript in Results and analysis section with blue color text, Page – 10, Line – (298-300).

2. What are the threshold values of the PPI network used in this paper?

Author response: Thank you for your comments. In PPI network, we analysis the whole network. For that we didn’t set any threshold value. While identification of hub genes we set a threshold value which was greater than 10.

Author action: According to your suggestion, we have revised and updated the manuscript in Results and analysis section with blue color text, Page – 10, Line – (294-295).

3. Few of the potential hub genes identified in this study require some validation using mRNA expression datasets obtained from PTB and NCD patient tissue samples.

Author response: Thank you for your thoughtful review of our manuscript. We appreciate your insightful suggestion regarding the potential validation of the identified hub genes using mRNA expression datasets obtained from PTB and (NCD) patient tissue samples.

While we acknowledge the importance of external validation, we have validated hub genes by RNA-seq datasets. To validate genes, we have used GEO dataset - PTB (GSE54992, GSE19442), PD (GSE20295, GSE22491), RA (GSE23561, GSE157047), CKD (SE66494, GSE15072, GSE141295), CVD (GSE51878, GSE141910), LC (GSE42826, GSE30219), and DM (GSE92724, GSE236746) from NCBI. To extract upregulated genes, where log-fold change (logFC) ≥ 0.5 and P-value and adj P-value < 0.05. To adjust P-value we have used Benjamini–Hochberg method.

Author action: According to your suggestion, we have revised and updated the manuscript in Introduction, Methodology and Results and analysis section with blue color text, Page- (3, 6, 8, 16-18), Line- (100-101, 170-171, 257-259, 260-273, 394-397), Reference – (80-94), and Table – (2, 8-14).

4. To enhance the visual understanding for the readers, it will be a good idea to represent the overlapping genes between PTB and NCDs in a Venn diagram instead of the tabular format shown in Table 3.

Author response: Thank you so much for your valuable comment and effort. We have removed the table and made a Venn diagram to represent the overlapping genes.

Author action: According to your suggestion, we have revised and updated the table in a Venn diagram in Results and analysis section, Page – 9, Figure – 3, Line – (283-284).

5. This manuscript contains numerous typos and grammatical errors which need to be addressed properly by the Authors.

Author response: Thank you for your comments. According to your suggestion, we have improved grammatical mistakes in this manuscript.

Author action: According to your suggestion, we have revised and updated the manuscript.

6. The authors touch upon this in their Introduction section, but further justification is required for choosing these 7 NCDs specifically.

Author response: Thank you so much for your valuable comment and effort. TB and NCDs can coexist and increase the risk of each other. TB can impair immune surveillance, increasing susceptibility to NCDs, which together account for two-thirds of worldwide mortality. Because Some NCDs are incurable, thus early discovery can save lives. LC can be curable if treated early. However, PD, CKD, RA, DM, and SL are incurable disorders. CVD is curable; nevertheless, long-term treatment is required.

Author action: According to your suggestion, we have revised and updated the manuscript in Introduction section, Page 3, Line – (72-76 and 82-87), Reference – 31, 35-42.

7. Briefly describe the limitations of this study and what steps can be taken to address those limitations.

Author response: Thank you so much for your valuable comment and effort. This study also has limitations. This research focuses only on bioinformatics analysis. Furthermore, we collect data from a single source to detect pharmacological components that may be affecting performance.

Author action: According to your suggestion, we have revised and update the manuscript in Discussion section, Page – 20, Line – (503-504)

**Minor Comments:**

8. Rewrite the Objectives section as:

This study aims to identify the drug components that can regulate abnormal gene expression in NCDs. The study will reveal hub genes, potential biomarkers, and drug components associated with hub genes through statistical measures. This will contribute to targeted therapeutic interventions.

Author response: Thank you so much for your valuable comment and effort. We agree with the assessment. We have carefully considered your suggestion and taken action.

Author action: According to your suggestion, we have updated the manuscript at Objectives section in Abstract, Page – 1

9. Rewrite the Results and Conclusions section as:

In this study, we found that all of the hub genes are linked with the 10 drug components, and it was observed that aspirin CTD 00005447 was mostly associated with all the other hub genes. This bio-informatics study may help researchers better understand the cause of PTB and its relationship with NCDs and eventually this can lead to exploring effective treatment plans.

Author response: Thank you so much for your valuable comment and effort. We agree with the assessment. We have carefully considered your suggestion and taken action.

Author action: According to your suggestion, we have revised and update the manuscript at Results and Conclusions section in Abstract, Page – 1

10. Page 4, line 119

Lifestyle, environmental factors, behaviors, and dietary information can cause non-communicable diseases.

Change ‘dietary information” to dietary intakes.

Author response: Thank you so much for your valuable comment and effort. We have updated the word according to your suggestion.

Author action: According to your suggestion, we have revised and updated the manuscript with blue color text in Related Work & Motivation section, Page – 4, Line – 144.

11. Please rewrite/modify the section containing the research gap (Introduction section Para The introduction should be concise and to the point.

Author response: Thank you for your review. According to your review, we have separated the Introduction into Introduction and Related work & motivation.

Author action: According to your suggestion, we have revised and divided the Introduction section into Introduction and Related work & motivation section, Page – 2, 4.

12. Page 4, line 122 Rewrite this sentence as” It might be challenging to determine whether or not the diseases are connected because it is difficult to make a straight cause-and-effect inference.”

Author response: Thank you so much for your valuable comment and effort. We have updated the line according to your suggestion.

Author action: According to your suggestion, we have revised and rewrite the sentence in Related Work & Motivation with blue color text, Page – 4, Line – (147-148)

13. Page 4, line 129 Rewrite this sentence as “some studies included a smaller and narrow dataset wherein the essential risk factors associated with PTB such as smoking, diabetes, socioeconomic status was not taken into consideration.”

Author response: Thank you so much for your valuable comment. We have rewritten the line based on your suggestion.

Author action: According to your suggestion, we have revised and rewrite the sentence with blue color text in Related Work & Motivation section, Page – 5, Line – (154-156)

14. Page 4 line 132, Rewrite this sentence “This study works with seven non-communicable diseases because, to the best of our knowledge, other studies only worked with four or five non-communicable diseases.”

In this study, we identified /uncovered most of the NCDs as the existing studies have failed to adequately report all of them.

Author response: Thank you for your thoughtful review of our manuscript. We have replaced that line with your suggested line.

Author action: According to your suggestion, we have rewritten the sentence with blue color text in Work & Motivation section, Page – 5, Line – (157-158)

15. Page 4 line 237 “The co-expression and Physical Interaction Network have been accomplished by using GeneMANIA plugin”

Missing keyword “prediction”

The co-expression and Physical Interaction Network prediction was accomplished by using GeneMANIA plugin

Author response: Thank you for your thoughtful review. We have added your suggested word.

Author action: According to your suggestion, we have revised and rewrite the sentence with blue color text in Methodology section, Page – 7, Line – (250-251)

16. Page 7, line 244 Rewrite this sentence “It includes gene sets 244 linked to drugs and small molecules based on data about how gene expression changes because of drugs” as: It includes gene sets that relate the drugs and small molecules with the gene expression data obtained upon drug administration.

Author response: Thank you for your thoughtful review. Based on your suggestion, we have updated that line.

Author action: According to your suggestion, we have revised and rewrite the sentence with blue color text in Methodology section, Page – 8, Line – (257-259)

17. Use the standard scientific nomenclature for gene/protein names throughout the manuscript.

Author response: Thank you so much for your valuable comment. We appreciate your insightful suggestion. The scientific name of common genes is already present in table 3, Page- (9-10). As your suggestion we are adding a supplementary file as well. Also below are the scientific name of the genes-

- HMGB1 = high mobility group box 1
- IL10 = interleukin 10
- IL17A = interleukin 17A
- IL18 = interleukin 18
- NLRP3 = NLR family pyrin domain containing 3
- CXCL8 = C-X-C motif chemokine ligand 8
- IFNG = interferon gamma
- TNF = tumor necrosis factor
- NFKB1 = nuclear factor kappa B subunit 1

Author action: According to your suggestion we are adding a supplementary file.

18. This paper “An Integrative Network Approach to Identify Common Genes for the Therapeutics in Tuberculosis and Its Overlapping Non-Communicable Diseases” (Alam et al., 2021) should be cited.

Author response: Thank you for your insightful comments. We have cited this paper in the manuscript in the introduction section.

Author action: According to your suggestion, we have revised and added this paper in introduction section with blue color text, Page – 3, Reference – 37.

19. Page 12, line 299 “Notably, pathways such as Immune System (R-HSA-168256), Cytokine Signaling In Immune System (R-HSA-1280215), and Interleukin-10 Signaling (R-HSA-678378) are also connected with highest number of genes from Reactome pathway”

As per the Reactome pathway analysis, the pathways connected with the highest number of genes as:

1. Signalling By Interleukins R-HAS-449147

2. Cytokine Signaling In Immune System (R-HSA-1280215),

3. Immune system R-HAS-168256

Author response: Thank you for your insightful comments. We have rewritten this line according to your suggestion.

Author action: According to your suggestion, we have revised and rewritten this sentence in Results and analysis section with blue color text, Line– (328-332), Page - 11.
